# Association of inflammatory cytokines with type 2 diabetes mellitus and diabetic nephropathy: a bidirectional Mendelian randomization study

**DOI:** 10.3389/fmed.2024.1459752

**Published:** 2024-11-07

**Authors:** Siyuan Song, Jing Ni, Yuqing Sun, Qiang Pu, Li Zhang, Qianhua Yan, Jiangyi Yu

**Affiliations:** ^1^Affiliated Hospital of Nanjing University of Chinese Medicine, Nanjing, China; ^2^Department of Endocrinology, Nanjing University of Chinese Medicine, Nanjing, China; ^3^Department of Endocrinology, Jiangsu Province Hospital of Chinese Medicine, Affiliated Hospital of Nanjing University of Chinese Medicine, Nanjing, China; ^4^Department of Endocrinology, Nanjing Jiangning Hospital of Chinese Medicine, Affiliated Jiangning Hospital of Chinese Medicine, China Pharmaceutical University, Nanjing, China

**Keywords:** Mendelian randomization analysis, bidirectional, inflammatory cytokines, type 2 diabetes mellitus, diabetic nephropathy, horizontal pleiotropy

## Abstract

**Objective:**

Previous observational studies have suggested associations between various inflammatory cytokines with type 2 diabetes mellitus and diabetic nephropathy. However, the causal association remains uncertain.

**Method:**

Summary statistics for type 2 diabetes mellitus and diabetic nephropathy were obtained from a publicly available genome-wide association study. Data on inflammatory cytokines were sourced from a genome-wide association study on protein quantitative trait loci. The inverse variance-weighted method was applied as the primary method for causal inference. MR-Egger, weighted mode, and weighted median method were employed as supplementary analyses. Sensitivity analyses were performed to detect heterogeneity and potential horizontal pleiotropy in the study.

**Result:**

Genetic evidence indicated that elevated levels of fibroblast growth factor 19 levels promoted the occurrence of type 2 diabetes mellitus, and increased concentrations of fibroblast growth factor 21 levels, C-C motif chemokine 19 levels, eotaxin levels, and interleukin-10 mitigated the risk of developing type 2 diabetes mellitus, while type 2 diabetes mellitus did not exert a significant influence on said proteins. Elevated levels of tumor necrosis factor ligand superfamily member 14 and TNF-related activation-induced cytokine were associated with an increased risk of diabetic nephropathy, and increased concentrations of interleukin-1-alpha and transforming growth factor-alpha were potentially correlated with a diminished risk of diabetic nephropathy. Sensitivity analyses further ensure the robustness of our findings.

**Conclusion:**

Mendelian randomization analysis highlights a causal association between inflammatory cytokines with type 2 diabetes mellitus and diabetic nephropathy, offering valuable evidence and reference for future research.

## 1 Introduction

Type 2 diabetes mellitus (T2DM) is a common chronic metabolic disease (1). Persistent hyperglycemia in the blood may cause multiple system complications, which may pose significant risks to the health and life span of patients. Diabetic nephropathy (DN) is a common microvascular complication of T2DM, affecting about 40% of patients with T2DM (2, 3). It is also a major cause of end-stage renal disease (ESRD). Despite advances in treatment, the molecular mechanisms underlying DN remain unclear, and current therapies, including angiotensin-converting enzyme inhibitors (ACEIs), angiotensin II receptor blockers (ARBs), and sodium-glucose cotransporter 2 inhibitors (SGLT-2i), are primarily symptomatic (4–6). The pathogenesis of DN involves multiple factors, including dysregulation of kidney signaling pathways that affect metabolism, hemodynamics, inflammation, and autophagy, ultimately leading to ESRD (7–10).

Immune response and inflammatory modulators are closely related to pancreatic β cell dysfunction and insulin resistance in patients with T2DM. Pro-inflammatory cytokines such as interleukin-1β (IL-1β) can inhibit insulin secretion and pancreatic β cell proliferation by increasing the transcription and secretion of chemokines (11). Inhibition of inflammatory factors in diabetic mice can effectively protect islet cells and delay the progress of hyperglycemia (12). Oxidative stress and inflammation are central to the progression of DN. Increased oxidative stress in DN arises from an imbalance between hyperglycemia-induced reactive oxygen species (ROS) production and the antioxidant defense mechanisms (13, 14). DN-induced hemodynamic or metabolic dysfunction triggers inflammatory processes, leading to the release of chemokines and pro-inflammatory cytokines, such as IL-1β, interleukin-6 (IL-6), interleukin-18 (IL-18), and adhesion molecules like vascular cell adhesion protein-1 (VCAM-1) and intercellular adhesion molecule-1 (ICAM-1) (15, 16). Furthermore, chemokine signaling recruits immune cells, including macrophages and T lymphocytes, which release additional pro-inflammatory mediators, exacerbating renal inflammation (17, 18). ROS not only cause oxidative tissue damage but also promote the aggregation of inflammatory cells, leading to the production of inflammatory cytokines, growth factors, and transcription factors linked to DN pathogenesis (19). The infiltration of inflammatory cells, such as lymphocytes, neutrophils, and macrophages, is a key driver of kidney damage in DN (20). The recruitment and differentiation of these immune cells are regulated by various inflammatory cytokines (6, 21, 22). For example, upon stimulation by ROS, NF-kB translocates to the nucleus, binds to NF-kB binding sites, and activates transcription. This leads to the release of adhesion molecules and pro-inflammatory factors, including monocyte chemoattractant protein-1 (MCP-1), ICAM-1, transforming growth factor-β1 (TGF-β1), IL-1β, IL-6, and tumor necrosis factor-α (TNF-α), which can also regulate ROS levels (23). High glucose (HG) can induce NF-kB receptor activators in podocytes, further promoting DN progression (24). The NOD-like receptor protein (NLRP) 3 inflammasome regulates inflammation by cleaving pro-inflammatory cytokines, such as pro-IL-1β and pro-IL-18, into their active forms (25). Bruton’s tyrosine kinase (BTK) is activated in the kidneys of DN patients, and its knockdown can reduce macrophage-induced inflammation in diabetic mice by inhibiting NLRP3 inflammasome activity (26). High-mobility group box 1 (HMGB1), a nuclear non-histone protein, can enter the nucleus through active secretion or passive release. Once in the cytoplasm, HMGB1 participates in immune responses. When released extracellularly, HMGB1 acts as a potent inflammatory mediator, either alone or as part of a pro-inflammatory cascade, stimulating the immune system (27). HMGB1 is regulated by ROS and the NLRP3 inflammasome, which facilitate its translocation from the nucleus to the cytoplasm. Once in the cytoplasm, HMGB1 binds to Toll-like receptor 4 (TLR4), activates NF-kB, and induces an inflammatory response (28). Overall, numerous studies demonstrate that oxidative stress and inflammation are interdependent processes that coexist within an inflammatory environment. Inflammatory cells release substantial amounts of ROS at inflammation sites, resulting in increased oxidative damage.

Current research indicates that inflammatory cytokines play a role in the treatment of T2DM and DN. In a long-term randomized controlled trial, it was observed that the plasma total FGF-21 level and biological activity level of patients with T2DM decreased (29). A cross-sectional study found that the serum concentration of hGDNF in patients with T2DM was lower than that in the control group, and the level of hGDNF in patients with poor blood sugar control was also significantly reduced (30). Zhang et al. (31) demonstrated that interleukin-17C (IL-17C) contributes to DN and suggested that inhibiting IL-17C could offer a therapeutic approach. Murakoshi et al. (32) found that IL-6 is essential for the proliferation of glomerular mesangial cells and the activation and expansion of B cells. As a pleiotropic cytokine, IL-6 has diverse effects on the body and directly impacts the inflammatory response in DN. Nakamura et al. (33) identified MCP-1 as an early marker of kidney function changes in DN, with its levels reflecting disease progression (34, 35). Additionally, research has shown that DN progression may be influenced by the activation of various signaling pathways mediated by inflammatory cytokines (36, 37) and other biomarkers (38, 39). However, the precise genetic impact of these cytokines on T2DM and DN remains unclear, underscoring the need for further investigation.

**Figure 1:**
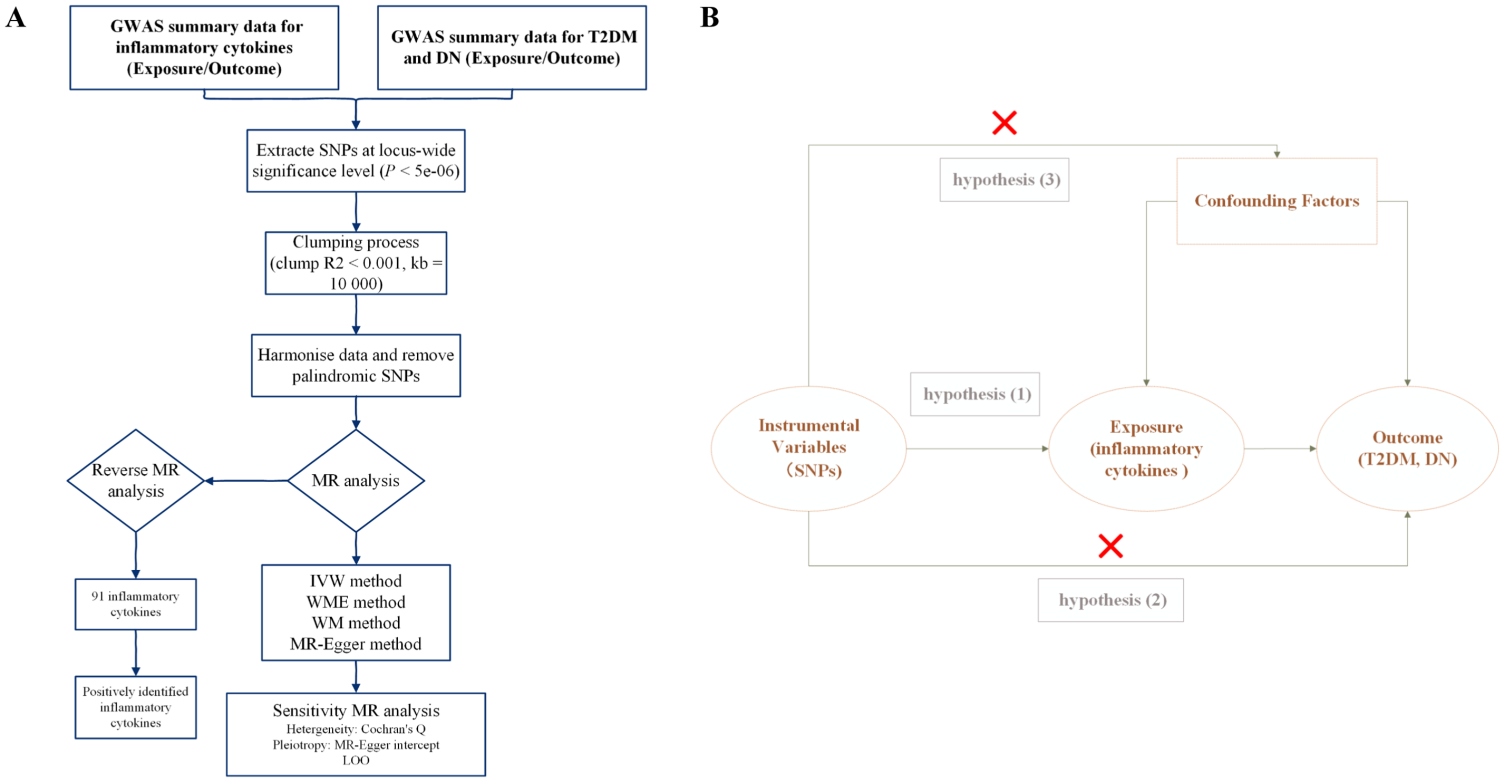
Study overview. (A) The protocol of our study procedure. T2DM, type 2 diabetes mellitus; DN, diabetic nephropathy; SNPs, single nucleotide polymorphisms; GWAS, genome-wide association study; LOO, leave-one-out; MR, Mendelian randomization; WM, weighted mode; WME, weighted median; IVW, inverse variance weighted. (B) The three hub hypothesis of the MR analysis.

Mendelian randomization (MR) analysis is a powerful method for elucidating causal associations between exposures and outcomes, utilizing SNPs as instrumental variables (IVs) (40, 41). Recently, MR has become a valuable tool for assessing these causal relationships (42). In our study, we employed a two-sample bidirectional MR analysis to examine the causal association between inflammatory cytokines with T2DM and DN, providing genetic evidence for this association. The procedural framework of our study is illustrated in Figure 1A.

## 2 Material and method

### 2.1 Exposure and outcome data sources

To estimate SNP effects associated with inflammatory cytokines, we used genome-wide association studies (GWAS) summary statistics (GSCT90274758–GSCT90274848) provided by Zhao et al. (43), which include data on 91 inflammatory cytokines from 14,824 individuals of European ancestry. Summary statistics for T2DM and DN were obtained from a publicly available GWAS analysis, which comprising 38,841 T2DM cases and 451,248 controls of European ancestry (ebi-a-GCST90018926), 1,032 DN cases and 451,248 controls of European ancestry (ebi-a-GCST90018832), 220 DN cases and 132,764 controls of East Asian ancestry (ebi-a-GCST90018612), sourced from the IEU OpenGWAS Project website.^1^ The dataset for T2DM European ancestry included 490,089 individuals and 24,167,560 SNPs (44). The dataset for DN European ancestry included 452,280 individuals and 24,190,738 SNPs, while the dataset for East Asian ancestry included 132,984 individuals and 12,447,074 SNPs (44).

Since this study relies on publicly available data, no additional ethical approval or consent was required. To minimize the impact of race-related confounding factors, the study population predominantly comprised individuals of European ancestry. Data from individuals of East Asian descent were also included to enhance the robustness and generalizability of the findings.

### 2.2 Instrumental variables selection

The selection of genetic variants as IVs for inflammatory cytokines followed the three-hub hypothesis (Figure 1B). First, SNPs were screened from the GWAS data, considering only those with a significance threshold of *p* < 5 × 10^−6^. A linkage disequilibrium test was then performed on these SNPs to ensure they met the independence criterion. SNP selection was carefully controlled with parameters set to *R*^2^ < 0.001 and a maximum distance of 10,000 kb to reduce linkage disequilibrium and identify independent SNPs (45). Second, the PhenoScanner database was used to validate whether the identified SNP loci were associated with other potential confounding factors (46). Finally, to evaluate the susceptibility of the selected SNPs to weak IV bias, *F* statistics were calculated with a threshold set at *F* > 10 (using the formula *F* = β^2^/SE^2^, where β represents the effect size and SE the standard error). SNPs with *F* < 10 were deemed susceptible to weak IV bias and excluded to avoid their influence on the results. Higher *F* statistics indicate that the genetic markers are likely to cause phenotypic variations, which can lead to divergence in results due to these variations (47, 48). The selection of genetic variants as IVs was associated with the exposure of inflammatory cytokines.

### 2.3 Statistical analysis

#### 2.3.1 MR analysis

To uncover the causal association between inflammatory cytokines with T2DM and DN, we employed a range of analytical methods, including inverse variance weighted (IVW), weighted median (WME), weighted mode (WM), MR-Egger regression, and forest plot visualization (49, 50). Using multiple analytical approaches aimed to enhance the robustness and reliability of our findings. A significance threshold of *p* < 0.05 was used to determine causal associations between inflammatory cytokines with T2DM and DN (45, 51).

To address the potential for specific errors and account for multiple testing, we applied the *q* value to adjust for the false discovery rate (FDR). A *q* value <0.1 indicates a significant association, while a *p* < 0.05 but *q* value ≥0.1 suggests a more tentative association (52).

#### 2.3.2 Sensitivity analysis

The *p*-value from Cochran’s *Q* statistics of the IVW method was used to assess the heterogeneity of the IVs. A *p*-value ≥0.05 indicates the absence of heterogeneity in the causal analysis (53, 54). Additionally, a funnel plot was employed as a visual tool to detect heterogeneity, with a symmetrical distribution of SNPs suggesting homogeneity in the results (55). Depending on the presence or absence of heterogeneity, either a random-effects model or a fixed-effects model was chosen.

Pleiotropy was assessed using MR-Egger regression, with the intercept of the MR-Egger regression in a scatter plot providing insights into potential pleiotropic effects (56, 57). Leave-one-out (LOO) analysis was also conducted to evaluate the stability of the results by identifying SNPs with significant influence when individually removed (58). The risk association between inflammatory cytokines with T2DM and DN was quantified using odds ratios (OR) and 95% confidence intervals (CI), with statistical significance defined as *p* < 0.05.

#### 2.3.3 Bidirectional MR analysis

A two-sample bidirectional MR analysis was conducted to explore the potential reverse causal association between T2DM, DN (exposure) and inflammatory cytokines (outcome). The procedural steps for this bidirectional MR analysis closely followed those outlined for the previous MR analysis.

#### 2.3.4 Statistical software

All MR analyses were conducted using R (version 4.3.1) with the TwoSampleMR package.

## 3 Results

### 3.1 Instrumental variables selection

**Figure 2:**
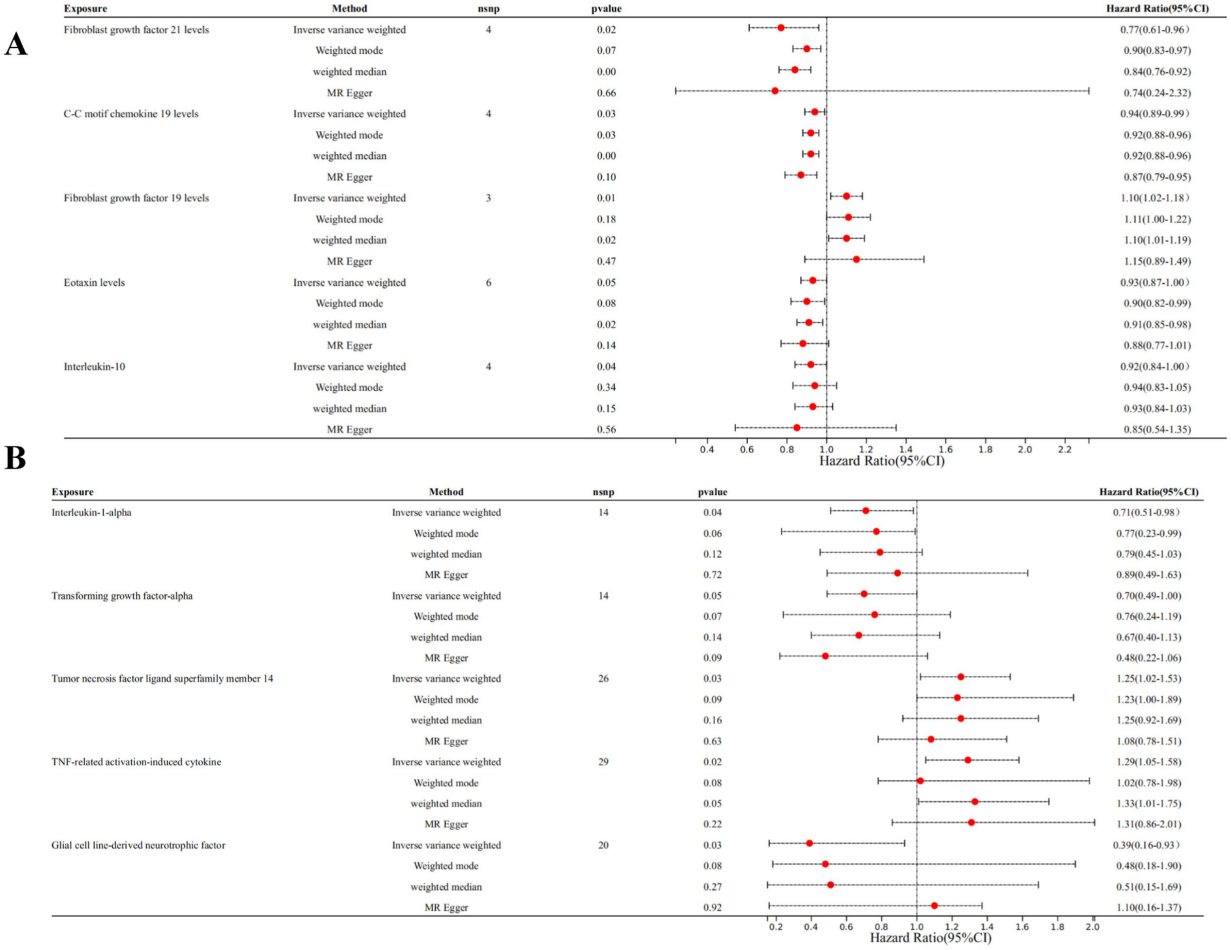
Forest plots of MR analysis. (A) Forest plot of the MR analysis results in T2DM dataset. (B) Forest plot of the MR analysis results in DN dataset. Exposure represents the inflammatory cytokines, *n*SNP represents the number of SNPs, and *p*val represents the *p*-value.

We selected 21 SNPs related to inflammatory cytokines in T2DM dataset, including 4 SNPs from Fibroblast growth factor 21 levels (FGF21), 4 SNPs from C-C motif chemokine 19 levels (CCL19), 3 SNPs from fibroblast growth factor 19 levels (FGF19), 6 SNPs from eotaxin levels (CCL11), 4 SNPs from Interleukin-10 (IL-10) (Figure 2A).

Among individuals of European ancestry in DN dataset, a total of 14 SNPs were extracted from tumor necrosis factor ligand superfamily member 14 (TNFSF14), 14 SNPs from TNF-related activation-induced cytokine (TRANCE), 26 SNPs from interleukin-1 alpha (IL-1α), and 29 SNPs from transforming growth factor alpha (TGF-α). For individuals of East Asian ancestry in DN dataset, 20 SNPs were extracted from glial cell line-derived neurotrophic factor (GDNF) (Figure 2B). Notably, the *F* statistics for the IVs used in this study exceeded 10, indicating minimal bias from weak IVs and ensuring the robustness of the results (Supplementary Table S1).

The substantial differences between European and East Asian populations stem from factors like genetic allele frequency variations, environmental factors, and lifestyle differences, which may result in distinct research outcomes. For instance, a particular SNP might be common in one population but rare in another. If racial differences are not considered, these frequency disparities could impact effect estimates in MR analyses, leading to biased conclusions. Beyond genetic differences, ethnic groups vary in terms of diet, lifestyle, culture, and socioeconomic status. These environmental distinctions could also influence outcomes through gene-environment interactions. To minimize race-related confounding, we focused primarily on individuals of European descent. However, data from those of East Asian ancestry were also included to improve the robustness and generalizability of our findings.

### 3.2 MR analysis

**Figure 3:**
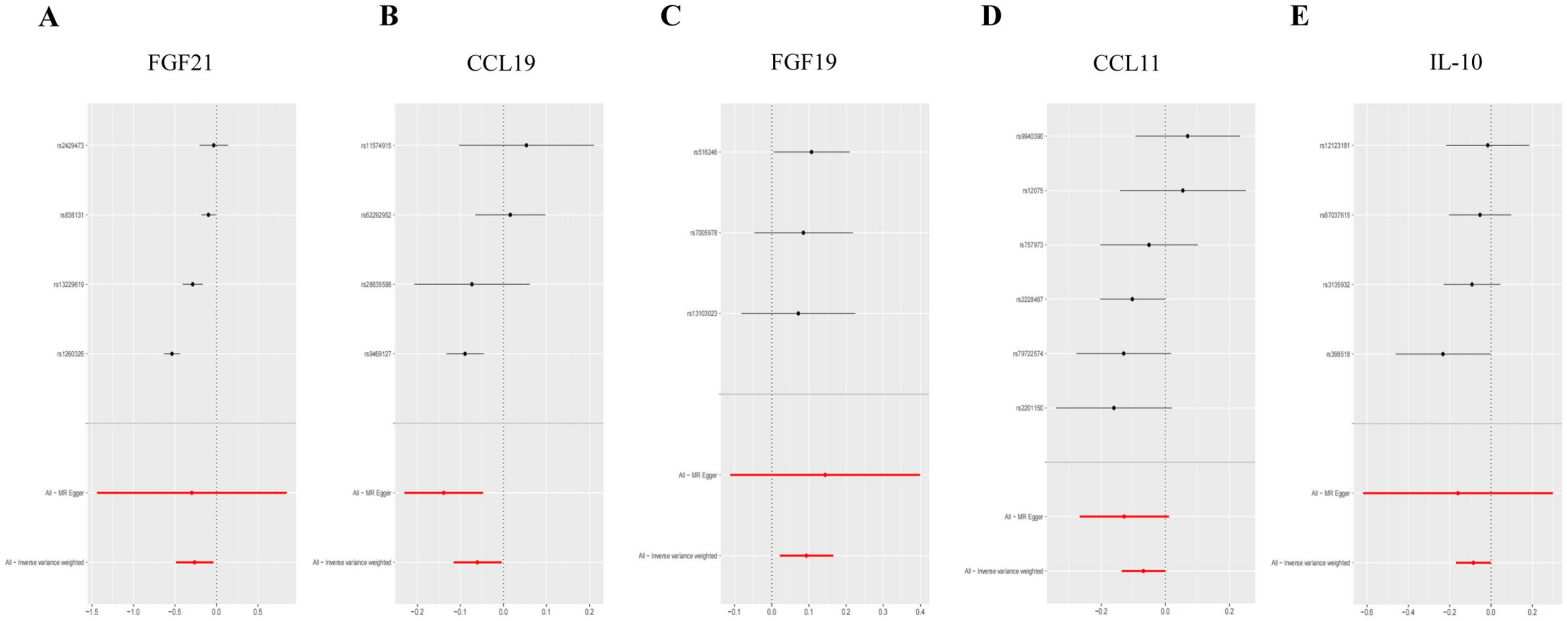
Forest plot of single SNP MR results in T2DM dataset. (A) Gene expression of FGF21. (B) Gene expression of CCL19. (C) Gene expression of FGF19. (D) Gene expression of CCL11. (E) Gene expression of IL-10. In this representation, each black dot symbolizes the T2DM with increased standard deviation (SD) in the inflammatory cytokine, generated by utilizing each SNP as an individual IV. Conversely, the red dot denotes the causal estimation derived from all SNP combinations using various MR methods. The horizontal line segment represents the 95% CI. Specifically, the IVW causal estimate illustrates how the overall estimate (depicted by the red horizontal line) might be disproportionately influenced by the removal of a single variant (indicated by the black horizontal line).

**Figure 4:**
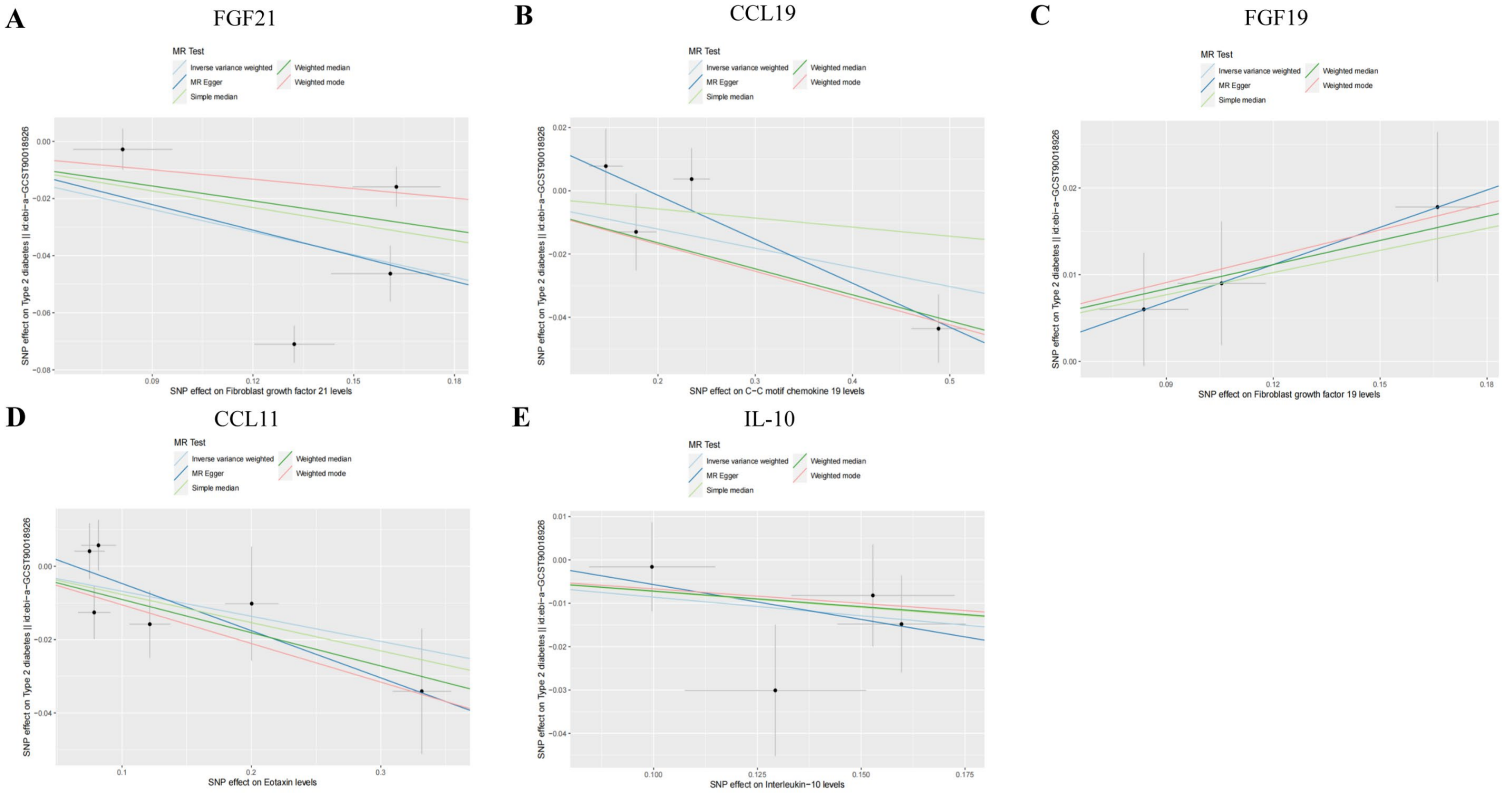
Scatter plots of SNP analysis in T2DM dataset. (A) Gene expression of FGF21. (B) Gene expression of CCL19. (C) Gene expression of FGF19. (D) Gene expression of CCL11. (E) Gene expression of IL-10. The *X*-axis denotes the impact of the SNP on the inflammatory cytokine, while the *Y*-axis represents the SNP’s influence on T2DM. Each black dot signifies a single SNP, with the line segment depicting the 95% CI. The slope of the straight line reflects the causal estimation derived from the MR method. In this visualization, the green line corresponds to the IVW method, the blue line represents the MR Egger method, the dark line signifies the WME method, the reseda green line represents the Simple median method and the red line represents the WM method.

Genetic evidence indicated that elevated levels of FGF19 levels (OR = 1.100, 95% CI 1.020–1.180, *p* = 0.030) promoted the occurrence of T2DM, and increased concentrations of FGF21 levels (OR = 0.770, 95% CI 0.610–0.960, *p* = 0.020), CCL19 (OR = 0.050, 95% CI 0.930–0.990, *p* = 0.030) levels, CCL11 levels (OR = 0.930, 95% CI 0.870–1.000, *p* = 0.050), and IL-10 (OR = 0.920, 95% CI 0.840–1.000, *p* = 0.040) mitigated the risk of developing T2DM (Figures 3, 4).

Among individuals of European ancestry in DN dataset, the IVW analysis revealed a significant positive causal association between the gene expression of TNFSF14 (OR = 1.249, 95% CI 1.018–1.532, *p* = 0.033) and TRANCE (OR = 1.287, 95% CI 1.051–1.577, *p* = 0.015) with DN. In contrast, gene expression of IL-1α (OR = 0.712, 95% CI 0.514–0.984, *p* = 0.040) and TGF-α (OR = 0.701, 95% CI 0.493–0.998, *p* = 0.049) showed a significant negative causal association with DN. Among individuals of East Asian ancestry in DN dataset, gene expression of GDNF was significantly negatively associated with DN (OR = 0.391, 95% CI 0.163–0.931, *p* = 0.034) (Supplementary Figure S1).

Given that all SNPs functioned as effective IVs without horizontal pleiotropy and that the IVW analysis results remained unbiased, the IVW method was considered more reliable for providing effect estimates compared to alternative methodologies (59). This supports the conclusion that inflammatory cytokines have a causal association with T2DM and DN.

However, after FDR correction, only FGF21 (*q* value = 0.001) and CCL19 (*q* value = 0.025) levels showed a significant causal association with T2DM, while the gene expression of IL-1α showed a significant negative causal association with DN (*q* value = 0.050). The associations for the other inflammatory cytokines were considered suggestive of a link to T2DM and DN.

### 3.3 Sensitivity analysis

**Figure 5:**
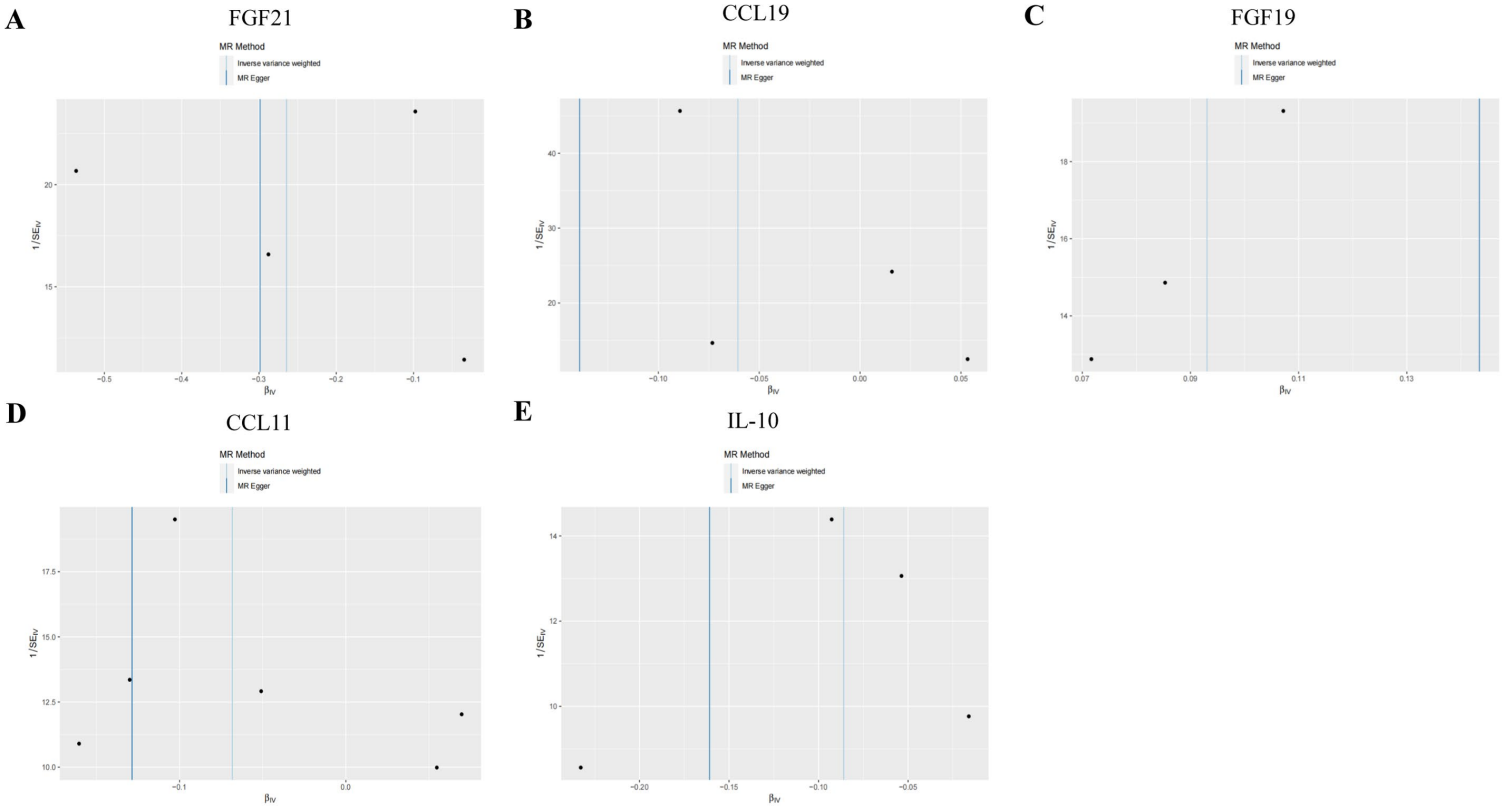
Funnel plots of sensitivity analysis in T2DM dataset. (A) Gene expression of FGF21. (B) Gene expression of CCL19. (C) Gene expression of FGF19. (D) Gene expression of CCL11. (E) Gene expression of IL-10. The light blue represents the IVW method, and the dark blue represents the MR Egger method.

**Figure 6:**
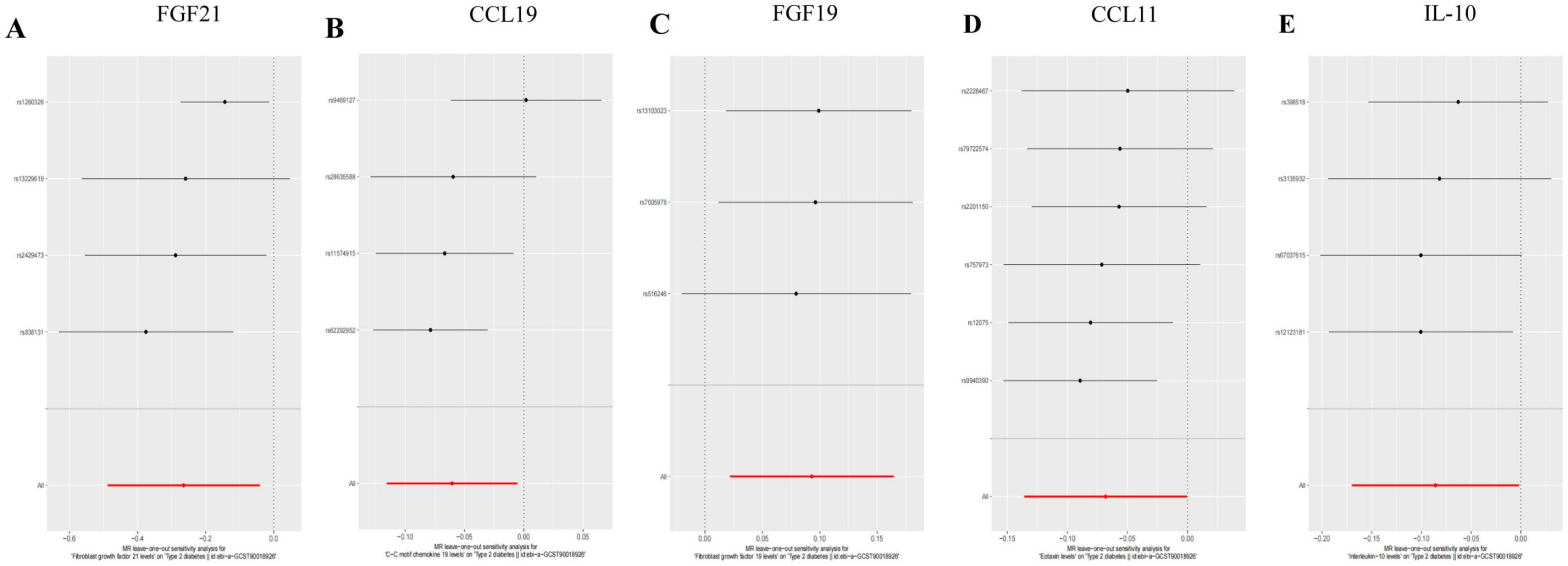
Forest plots of LOO analysis in T2DM dataset. (A) Gene expression of FGF21. (B) Gene expression of CCL19. (C) Gene expression of FGF19. (D) Gene expression of CCL11. (E) Gene expression of IL-10. In this representation, each black dot symbolizes the T2DM with increased standard deviation (SD) in the inflammatory cytokine, generated by utilizing each SNP as an individual IV. Conversely, the red dot denotes the causal estimation derived from all SNP combinations using various MR methods. The horizontal line segment represents the 95% CI. Specifically, the IVW causal estimate illustrates how the overall estimate (depicted by the red horizontal line) might be disproportionately influenced by the removal of a single variant (indicated by the black horizontal line).

In both T2DM and DN datasets, Cochran’s *Q* test revealed no evidence of heterogeneity among the included IVs (*p* > 0.05). Additionally, the MR-Egger regression intercept test indicated that pleiotropy did not bias the results (*p* > 0.05) (Supplementary Table S2). Funnel plots visually confirmed that potential confounders were unlikely to affect the causal inferences (Figure 5; Supplementary Figure S2). The LOO sensitivity analysis showed that removing individual SNPs did not significantly alter the analysis results (Figure 6; Supplementary Figure S3).

### 3.4 Bidirectional MR analysis

**Figure 7:**
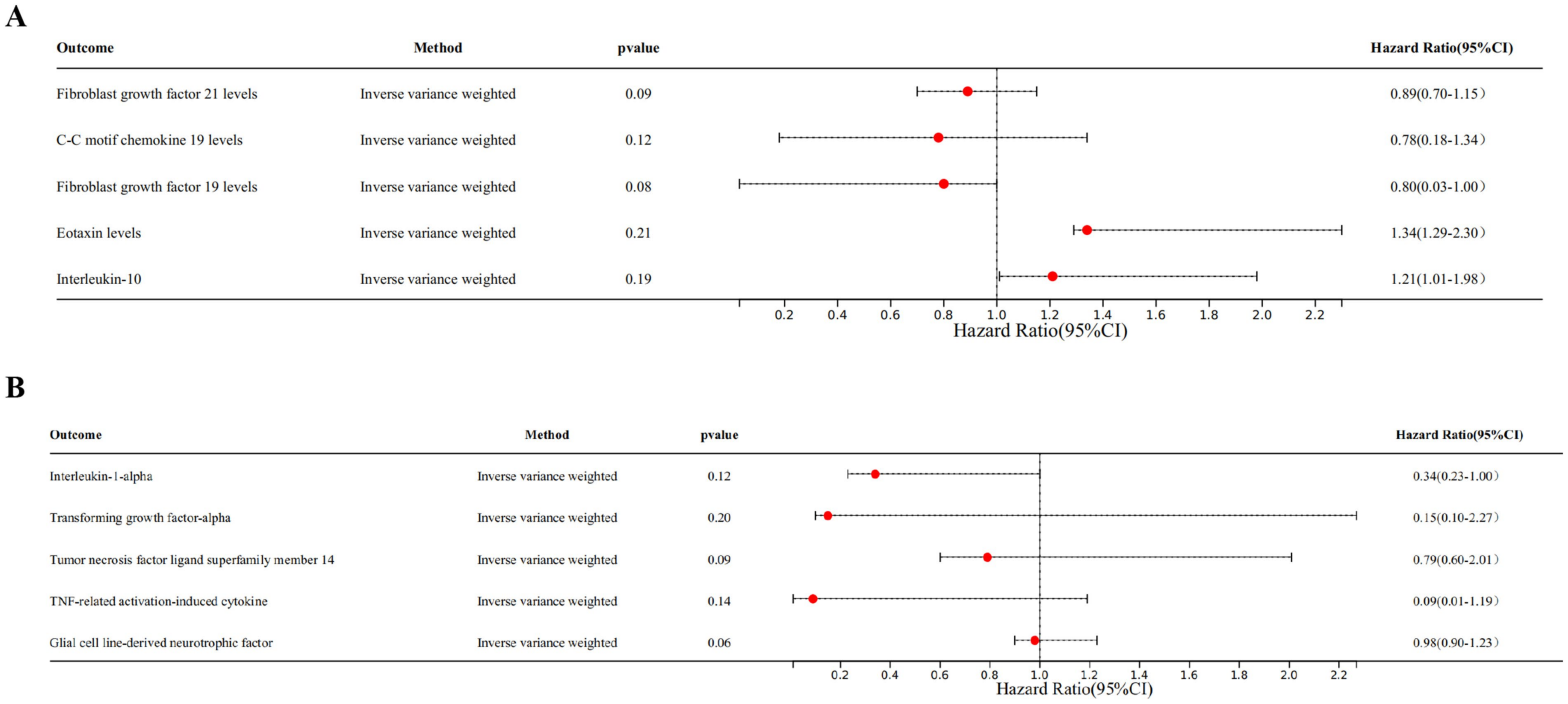
Forest plots of bidirectional MR analysis. (A) Forest plot of the MR analysis results in T2DM dataset. (B) Forest plot of the MR analysis results in DN dataset. Outcome represents the positive identified inflammatory cytokines, *n*SNP represents the number of SNPs, and *p*val represents the *p*-value.

The reverse MR analysis revealed there is no causal association between T2DM, DN and the positive inflammatory cytokines previously identified (Figures 7A,B).

## 4 Discussion

In this study, we conducted a comprehensive analysis using large-scale GWAS data to investigate the causal association between inflammatory cytokines with T2DM and DN. To address the three hypotheses, we first included only SNPs that met the criteria of *p* < 5 × 10^−6^, *R*^2^ < 0.001, and a maximum distance of 10,000 kb. Second, the PhenoScanner database was used to exclude potential confounding factors. Finally, only SNPs with *F* > 10 were included in the analysis. Genetic evidence indicated that elevated levels of FGF19 levels promoted the occurrence of T2DM, and increased concentrations of FGF21 levels, CCL19 levels, CCL11 levels, and IL-10 mitigated the risk of developing T2DM, while T2DM did not exert a significant influence on said proteins. Elevated levels of TNFSF14 and TRANCE were associated with an increased risk of DN, and increased concentrations of IL-1α and TGF-α were potentially correlated with a diminished risk of DN. Subsequent sensitivity analyses confirmed the robustness and consistency of these findings.

FGF19 is primarily secreted by the ileum and functions as an endocrine factor. It plays a crucial role in inhibiting bile acid synthesis and regulating hepatic metabolism (60). Previous studies have suggested that FGF19 may be involved in the regulation of glucose and lipid metabolism, as well as the maintenance of energy homeostasis. Additionally, it may play a protective role in preserving β-cell function (61). Zhang et al. (62) demonstrated in their study that FGF19 levels in subjects with normal glucose tolerance were significantly lower than those in subjects with impaired fasting glucose or impaired glucose tolerance. Moreover, when compared to patients with T2DM, FGF19 levels were markedly reduced in T2DM patients with metabolic syndrome. Human studies have found that serum FGF19 levels are lower in patients with T2DM compared to control groups. However, after weight loss induced by partial gastrectomy or gastroenterostomy, FGF19 levels increase, accompanied by improvements in glucose metabolism indicators (63). Previous studies have indicated that FGF19 acts as a protective factor against T2DM. However, the MR results suggest that FGF19 may serve as a risk predictor for T2DM. This discrepancy could be influenced by various factors, such as environmental conditions and innate genetic variations.

FGF21 is a key regulator of endocrine metabolism. It promotes glucose uptake in skeletal muscle by activating the phosphoinositide 3-kinase/protein kinase C (PI3K/PKC) signaling pathway, which mediates the translocation of glucose transporter 4 (GLUT4) (64). Additionally, FGF21 enhances glucose uptake in adipose tissue through the activation of the extracellular signal-regulated kinase 1/2 (ERK1/2) signaling pathway, thereby helping to maintain glucose and lipid homeostasis (65). FGF21 also induces the nuclear translocation of nuclear factor erythroid 2-related factor 2 (Nrf2), promoting the expression of antioxidant genes, which alleviates oxidative stress and improves insulin resistance (66).

CCL19 is a chemokine that plays a role in immune cell migration and inflammatory responses. Its relationship with the development of T2DM is linked to its involvement in chronic inflammation, a key factor in the pathogenesis of T2DM. In individuals with T2DM, persistent low-grade inflammation contributes to insulin resistance, pancreatic β-cell dysfunction, and metabolic disturbances (67). CCL19 is elevated in patients with T2DM and may promote the infiltration of immune cells into tissues such as adipose tissue, pancreas, and liver. This can exacerbate local inflammation and insulin resistance, contributing to the progression of T2DM. Moreover, CCL19 has been studied in relation to its effects on endothelial dysfunction and atherosclerosis, which are common complications of T2DM (68). The chemokine may accelerate vascular inflammation, increasing the risk of cardiovascular diseases in patients with diabetes. CCL19 is associated with T2DM through its role in promoting inflammation, contributing to insulin resistance, and possibly aggravating cardiovascular complications. CCL11 also known as eotaxin-1, is a chemokine primarily involved in the recruitment of eosinophils and other immune cells to sites of inflammation. Its relationship with T2DM has been increasingly recognized in recent years (69). CCL11 contributes to the development and progression of T2DM by enhancing inflammatory responses and promoting insulin resistance. A meta-analysis revealed that the expression levels of CCL11 are significantly higher in patients with T2DM compared to control groups (70). The upregulation of CCL19 and CCL11 have been confirmed in both T2DM datasets and high-glucose *in vitro* experiments. However, the MR results indicate that CCL19 and CCL11 act as a protective factor in T2DM, which may be attributed to genetic variations.

IL-10 is a multifunctional inflammatory cytokine that primarily regulates the body’s inflammatory response and immune balance through mechanisms such as humoral immune stimulation (71). It is an inhibitory cytokine that can attenuate the apoptosis of β-cells, suppression of insulin secretion, and peripheral insulin resistance induced by pro-inflammatory cytokines such as IL-6 and TNF-α. By improving pancreatic signaling pathways, IL-10 helps reduce the risk of developing T2DM (72).

TNFSF14 is involved in promoting renal fibrosis (73). IL-1 is pivotal in immune regulation and inflammatory response orchestration. Elevated serum levels and gene expression of IL-1 are consistently observed in patients with DN, which correlates with studies linking IL-1 gene polymorphisms to increased risk of end-stage DN (74, 75). Clinical studies have demonstrated that inhibiting EGFR with TGF-α monoclonal antibodies significantly delays the onset and progression of DN (76). Experimental evidence shows that TNF-α induces the production of inflammatory mediators, including prostaglandins, leukotrienes, and IL-1, in cultured human mesangial cells. These mediators are implicated in the pathogenesis of DN (77). Diabetic mice exhibit decreased GDNF levels, but local administration or overexpression of the GDNF gene in these mice has been shown to reduce apoptosis, improve β-cell quality and proliferation, enhance insulin secretion, and improve local tissue function. Despite these benefits, the efficacy of GDNF in blood sugar control remains a topic of debate (78, 79).

The inflammatory cytokines identified in this study not only a unique risk for DN, but also associated with other systemic diseases. For example, Huang et al. (80) found that TNFSF14 mediates the impact of docosahexaenoic acid on atopic dermatitis. Yan et al. (81) found that a decrease in IL-1α levels has been associated with the development of intervertebral disc degeneration. TGF-α holds diagnostic value in various diseases, including endometrial cancer (82) and non-small cell lung cancer (83). Its expression levels can serve as a biomarker, aiding in the early detection and diagnosis of these malignancies. However, there is still a lack of MR research evidence linking these inflammatory cytokines to systemic diseases. Future studies are needed to further investigate these associations and establish more robust causal relationships.

Unlike previous MR analyses that explored the relationship between immune cells or inflammatory cytokines and DN, this study stands out in several ways. First, we applied a more stringent threshold of *p* < 5 ×10^−6^, whereas others set it at 1 ×10^−5^, which led to a more selective identification of instrumental variables and a lower risk of pleiotropy. Second, this study assessed 91 inflammatory cytokines, as opposed to the 41 analyzed in earlier research. Third, we used a larger GWAS dataset with a greater number of cases, and we validated our findings using other ethnic GWAS datasets, ensuring the robustness and lack of bias in our results. Fourth, differences in the exposure and outcome datasets led to conclusions that contrast with those of prior studies. Fifth, a FDR correction was implemented, reducing the probability of false positives in the MR results. Additionally, a bidirectional MR analysis was carried out to reliably estimate the causal connection between exposure and disease, thus addressing the reverse causality issue often seen in traditional observational research. Finally, the PhenoScanner database was employed to eliminate potential confounders related to DN. However, certain limitations need to be acknowledged. Firstly, the outcome data originate from European and East Asian populations, other populations should be validated using local data. Secondly, the lack of detailed information, such as type, severity, and duration of diabetes, hinders the possibility of conducting further subgroup analyses. Therefore, they must be validated in future large-sample clinical trials. Thirdly, while MR facilitates the evaluation of the enduring impacts of genetically predisposed inflammatory cytokines throughout an individual’s lifespan, it may not directly encapsulate the attenuation of these factors in adulthood due to the influence of diverse unreported regulators. Fourthly, while we employed various procedures in MR to effectively exclude confounders, there remain potential confounding factors that may affect the study’s accuracy. For example, certain genetic variants can simultaneously influence multiple phenotypes (84). Additionally, for inflammatory factors that did not show statistical significance in our analysis, it is still possible that a causal relationship with DN or T2DM exists, which may not have been detected due to the limited SNPs available for these inflammatory factors (85). Finally, to conduct sensitivity and horizontal pleiotropy analyses, more SNPs needed to be included as IVs, so instead of the traditional significance threshold (*p* < 5 ×10^−8^), we chose 5 ×10^−6^.

## 5 Conclusion

This study revealed pivotal contributions of FGF19, FGF21, CCL19, CCL11, and IL-10 in the advancement of T2DM. The importance of TNFSF14, TRANCE, IL-1α, TGF-α, and GDNF in the progress of DN. These findings offered favorable implications for the treatment and prevention of T2DM and DN, laying the groundwork for novel clinical approaches and management strategies. However, additional experimental and clinical investigations are necessary to elucidate the functions and molecular mechanisms of these inflammatory factors in future studies.

## Data Availability

The original contributions presented in the study are included in the article/Supplementary material, further inquiries can be directed to the corresponding authors.

## Footnotes

^1^ https://gwas.mrcieu.ac.uk
