# Supplementary figures and images for: Association of inflammatory cytokines with type 2 diabetes mellitus and diabetic nephropathy: a bidirectional Mendelian randomization study
Source: Front Med (Lausanne). 2024 Nov 7;11:1459752. doi: 10.3389/fmed.2024.1459752 (PMC11580751; doi:10.3389/fmed.2024.1459752)

Supplementary Figure 1 Scatter plots of SNP analysis in DN dataset

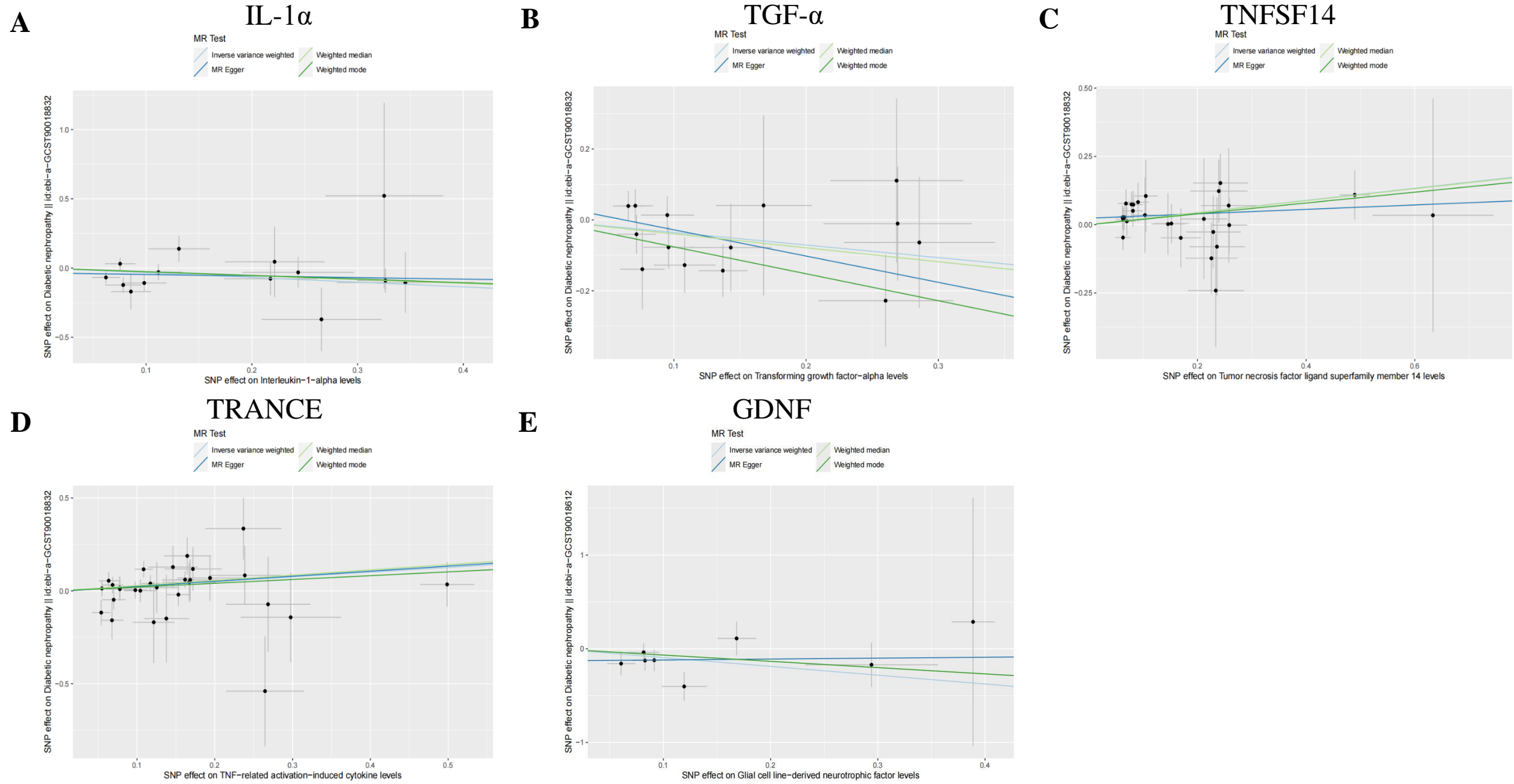

Supplement: Supplementary file 3 [file Data_Sheet_1.PDF]

Supplementary Figure 2 Funnel plots of sensitivity analysis in DN dataset

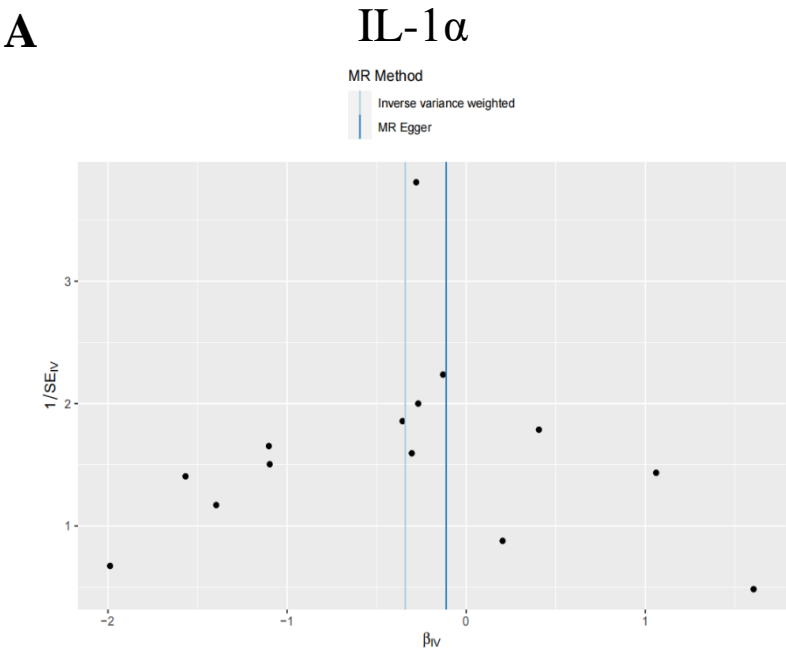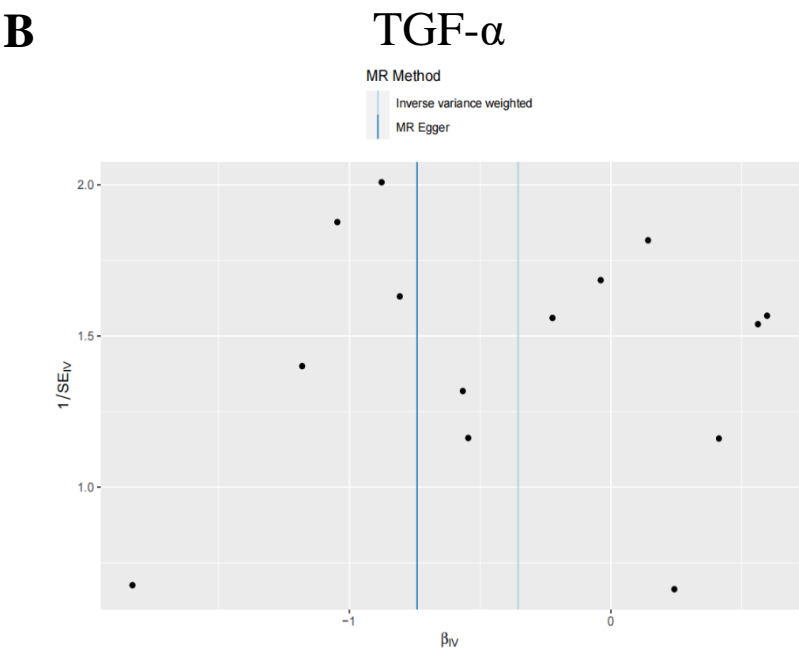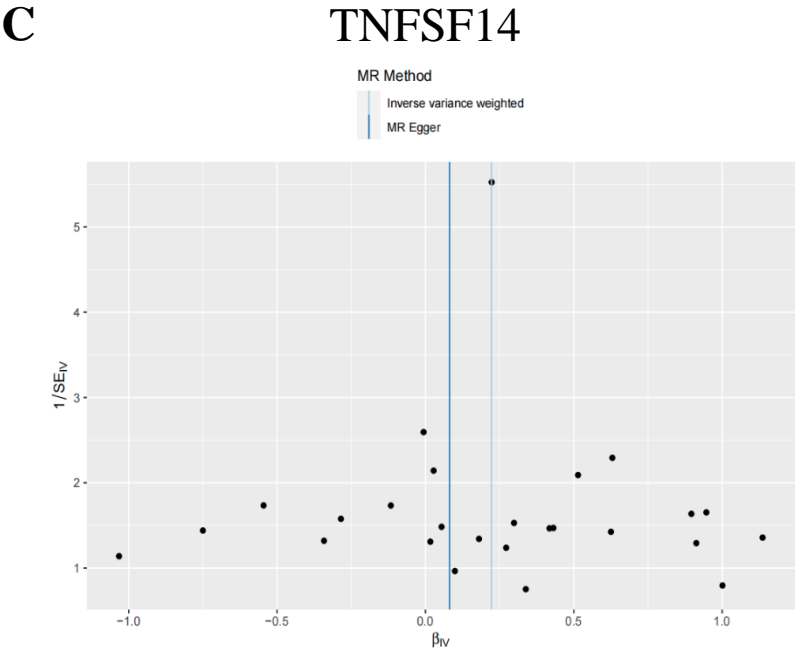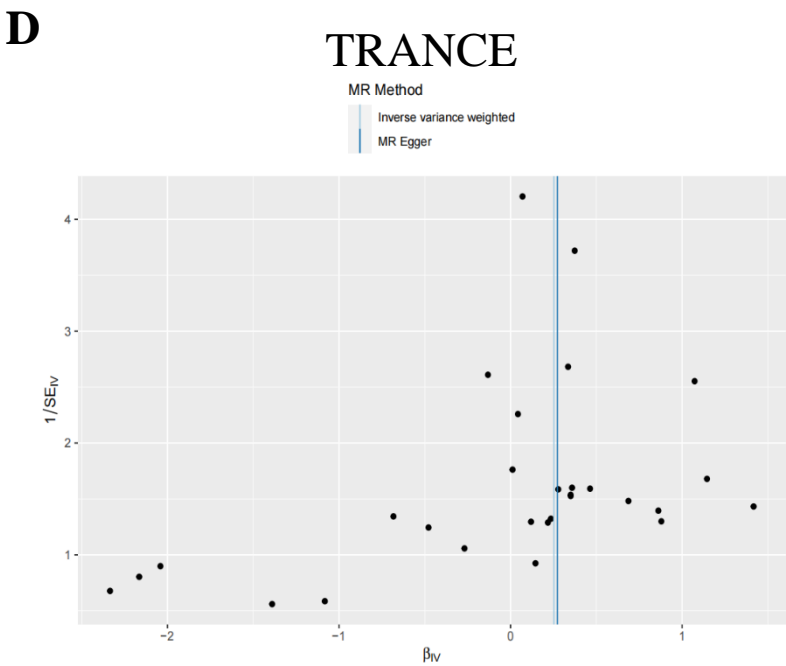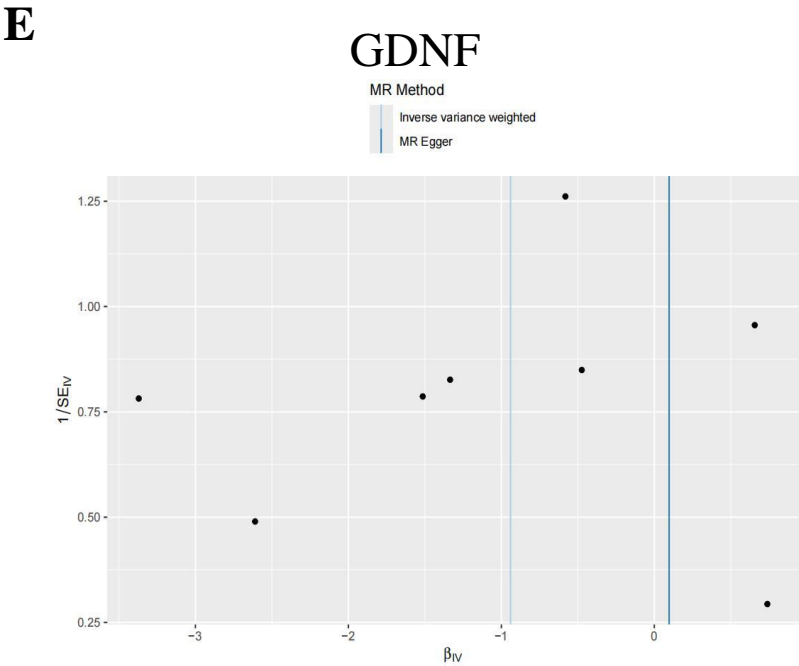

Supplement: Supplementary file 4 [file Data_Sheet_2.PDF]

Supplementary Figure 3 Forest plots of LOO analysis in DN dataset

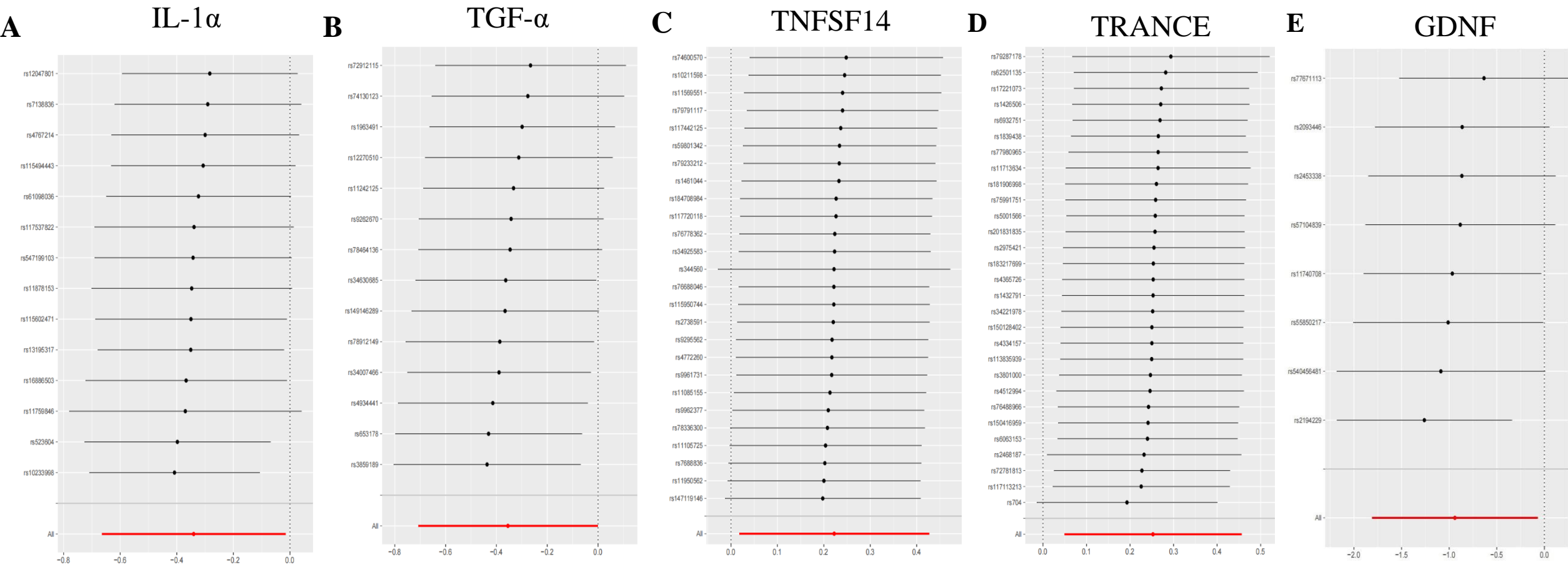

Supplement: Supplementary file 5 [file Data_Sheet_3.PDF]
